# From transcriptional regulation to drugging the cancer epigenome

**DOI:** 10.1186/s13073-014-0123-1

**Published:** 2014-12-20

**Authors:** James E Bradner

**Affiliations:** Department of Medical Oncology, Dana-Farber Cancer Institute, and Department of Medicine, Harvard Medical School, Boston, MA 02115 USA

## Abstract

Jay Bradner discusses the opportunities and challenges for the study and therapeutic targeting of the cancer epigenome, as well as innovative approaches to drug discovery.

## Introduction

**Figure 1:**
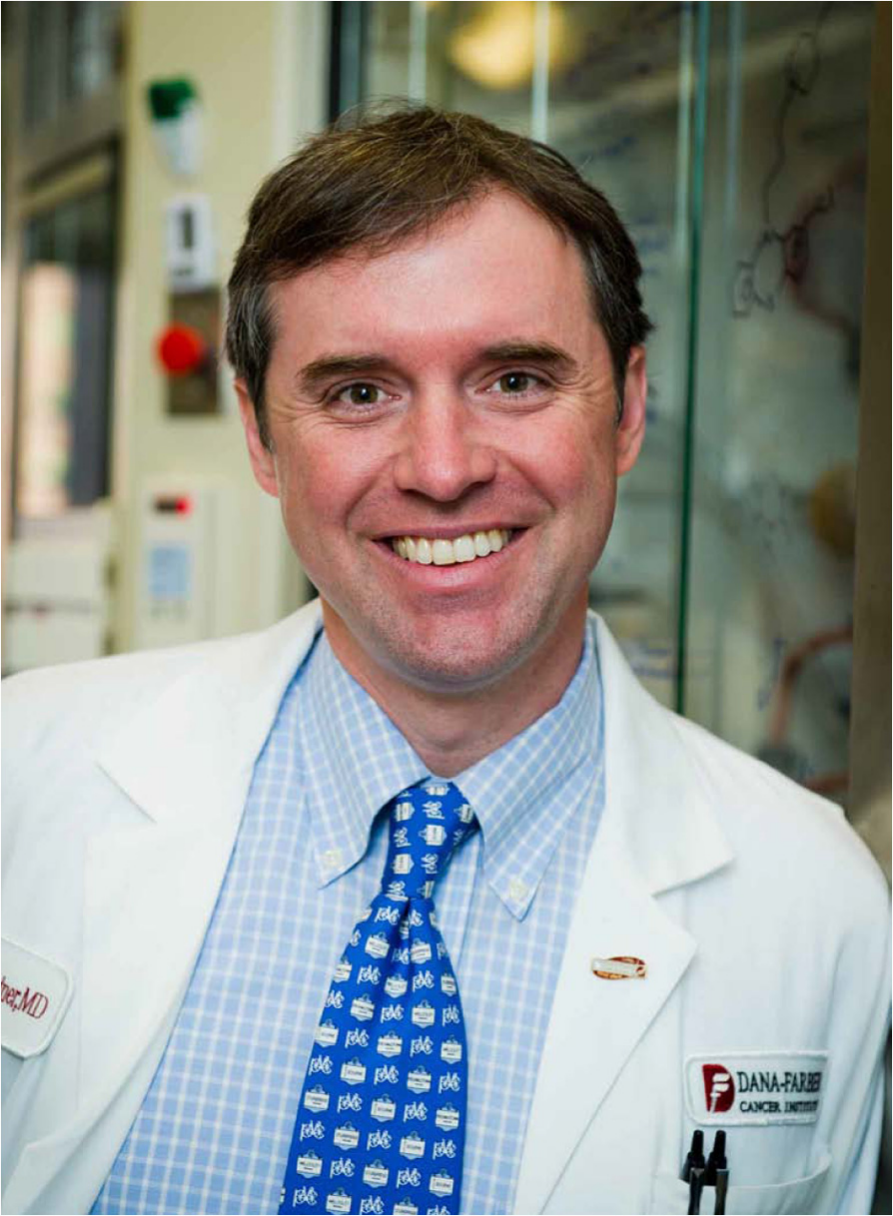
**Jay Bradner.**

Jay Bradner (Figure 1) is an Associate Professor in the Department of Medical Oncology at the Dana-Farber Cancer Institute and the Department of Medicine at Harvard Medical School. Additionally, Dr Bradner is the Associate Director for the Center for the Science of Therapeutics at the Broad Institute. The Bradner laboratory studies gene regulatory pathways, using chemical biology approaches such as the development of new chemical probes and innovative technologies to understand the role of chromatin in cancer. Already, their study of cancer gene regulatory pathways with chemistry has translated three first-in-class molecules to human clinical investigation as investigational cancer therapeutics.

## How did your interest in transcriptional regulation and epigenomics begin?

I became interested in gene regulation at Harvard College. I worked in three laboratories during my undergraduate education in biochemistry, with Profs Mark Ptashne, Bruce Demple and Thomas O’Halloran (Northwestern University). Each studied transcriptional biology, with nuanced differences in focus. Each was a wonderful environment to be exposed to the rigor and scholarship of basic research. In those early years, we were interested in the allostery of heavy metals that bind the transcription factors, sense toxins or environmental changes such as mercury or superoxide, and effect novel mechanisms of transactivation. Many years later, now in my own independent laboratory, we continue to study transcription, but in human cells. We are particularly interested in how chemical modulation of chromatin structure might influence chromatin-dependent transcriptional signaling.

For sure, my laboratory’s present focus in transcriptional biology was influenced by the fortune of being exposed to serious, mechanistic, basic biology at an early age. A disease-specific interest in cancer emerged through my subsequent training as an oncologist. Curious about mechanisms of disease pathogenesis and the pharmacopoeia, I attended the Pritzker School of Medicine at the University of Chicago. There, and since then, it has become apparent that cancer is fundamentally a heterogeneous disease of heterogeneous genetic alterations, the sum of which lead to homogenous deregulation of a short list of master regulatory transcription factors. Both the most frequently altered tumor suppressor in cancer (*TP53*), and the most frequently activated oncogene (*MYC*), are master regulators of cell growth and survival. In fact, almost all cancer growth signaling pathways converge on *MYC*, which to date still lacks direct-acting therapeutics. So we are highly motivated to understand the function of *MYC* at regulatory regions, and the deregulation of *MYC* via its own regulatory elements.

## Why do you think this area of research, of drugging the cancer epigenome, is gaining importance right now?

The field of epigenomics and transcriptional biology is just exploding at the moment. It is a very exciting time, when the basic biology and biochemistry of chromatin structure and function can be appreciated at the genome scale, but also with atomic resolution, through advances in biochemistry and structural biology. Additionally, the centrality of these altered transcriptional pathways in cancer are clear from genome sequencing studies, which have categorically identified alterations in gene regulatory proteins in nearly every type of human cancer. My back of the envelope analysis of the somatic alterations in cancer suggests that as many as 40% to 45% of all genes altered in cancer are gene regulatory factors, and *MYC* alone may be somatically altered in more than 40% of aggressive malignancies [1].

This convergence has created an opportunity to develop first-in-class molecules that disrupt these altered transcriptional pathways. Innovations in protein and cellular biochemistry arising from academia and within the commercial sector have created powerful platform capabilities to discover and optimize target-directed agents. We have found genome-wide measurements of chromatin structure, enhancer factor localization and RNA Polymerase II response to be very powerful in understanding, contrasting and positioning new chemical entities targeting chromatin-associated proteins.

Importantly, prototypical small molecules targeting the so-called ‘readers’, ‘writers’ and ‘erasers’ of chromatin are rapidly progressing through follow-up chemistry, receiving guidance from chemical biology and epigenomic research, and translating to human clinical investigation.

So this is no longer aspirational. In the last 2 years, we have already observed meaningful early activity among several distinct classes of epigenome-targeted agents in various solid and liquid forms of cancer. Hopefully for our patients, this renaissance of chromatin biology will bring a revolution in cancer medicine.

## What epigenetic drugs have reached the clinic or are in clinical development so far?

Small-molecule modulators of transcriptional pathways have been in use in the clinic for many years. Tamoxifen (targeting the estrogen receptor in breast cancer), bicalutamide (targeting the androgen receptor in prostate cancer) and all-*trans* retinoic acid (targeting the retinoic acid receptor fusion in acute promyelocytic leukemia) have been among the most meaningful anticancer agents for many years. These molecules bind and disrupt the function of transcription factors, firmly validating transcriptional therapy in cancer. However, significant challenges exist in discovery chemistry when moving beyond factors such as these that possess ligand-binding domains.

We therefore seek to develop molecules that modulate accessory factors, known or previously unrecognized, that are mechanistically required for master regulatory transcription factor function. The field has already produced US Food and Drug Administration-approved drugs targeting chromatin-associated enzymes, such as DNA methyltransferases (for example, azacitidine, Celgene, New Jersey, USA; and decitabine, MGI Pharma, Minnesota, USA), and histone deacetylases (for example, Vorinostat, Merck Research Laboratories, New Jersey, USA; Romidepsin, Celgene). The historic ease of targeting enzymes with small molecule therapeutics has recruited significant effort to inhibit the writers and erasers of chromatin, such as lysine methyltransferases and lysine demethylases, respectively. This second wave of small molecules includes inhibitors of the DOT1L and EZH2 lysine methyltransferases, inhibitors of the LSD1 lysine-specific demethylase, and inhibitors of the isocitrate dehydrogenase 2 enzyme (IDH2, a metabolic enzyme which when mutated causes the production of high concentrations of an epimetabolite that modulates chromatin structure).

Broadly speaking, the majority of transcription factors function by protein-protein interaction, and abrogating such interactions has proven challenging in the discipline of ligand discovery. As an academic group, we were comfortable approaching this challenge and created the first inhibitors of epigenomic ‘reader’ proteins. Bromodomain-containing proteins recognize acetylated lysine in active regions of transcription, recruiting other co-activator proteins to enforce transcription, as we have learned, of master regulatory transcription factors such as MYC. By displacing the BET family of bromodomains with a first direct-acting small-molecule inhibitor, JQ1, MYC transcription is impaired and MYC-addicted cancer cells die, senesce or terminally differentiate. We hope this research opens a new avenue of transcriptional drug discovery.

Overall, there is a rich pipeline of molecules in this class at various stages of preclinical development, suggesting that over the next decade this research – now in the clinic – will define the scope and impact of targeting epigenomic pathways in cancer.

## Which of these strategies do you think would be the most promising for epigenetic cancer therapy?

This is a very difficult question for the broader research community to answer in the fullness of time. I do believe, as an academic chemical biologist, that empowering the community with high-quality small-molecule probes will allow directed science to tease out the killer opportunities for definitive therapeutic development. With that said, these drugs must be developed wherever somatic alterations of the target oncogene exist. IDH2 inhibitors are naturally being developed for IDH2-mutant leukemia, EZH2 inhibitors are being studied in EZH2-altered B-cell lymphoma, and our BET inhibitors are actively being studied in BET-rearranged lung cancer and BET-rearranged head and neck cancer (so-called NUT midline carcinoma). I would also advocate for the development of these new compounds in context-specific dependencies, such as DOT1L inhibition with MLL-rearrangement in pediatric leukemia or BET inhibition with MYC or MYCN addiction.

## What are the main challenges in drugging the cancer epigenome as opposed to other molecular targets?

A number of challenges exist. First, transcriptional targets reside in the nucleus, which restricts the use of therapeutic technologies significantly. Immunoglobulins, peptides and nucleic acids lack the delivery properties to target epigenomic pathways with the efficiency of delivery required for a cancer therapeutic. Therefore, a major opportunity exists in the science of therapeutics to develop classes of agents and modes of delivery that would expand the arsenal of technologies available for disrupting gene regulatory pathways.

Second, gene regulatory pathways function largely via macromolecular assembly. Interfacial protein binding surfaces are often extensive, lacking the hydrophobic invaginations into which small molecules efficiently bind. Our positive experience targeting bromodomain-histone interactions argues that disrupting transcriptional complexes with high ligand efficiency is feasible, and therefore argues for the detailed functional and structural dissection of biophysical assemblies for sites of interaction well-suited to small-molecule discovery chemistry. In truth, these challenges are conceptual. Groups comfortable with assuming the risk and horizon of research required to develop transcriptional inhibitors will find success.

Third, we need much better measurements to guide the use of these therapies. Where tumors possess putative oncogenic drivers as somatic alterations, it is compelling to study small molecules that target these oncogenic alleles in patients that possess them. This is now obvious. The development of small molecules targeting epigenomic pathways will naturally benefit from new types of epigenomic biomarkers, such as for measurements of chromatin structure and function that confirm target engagement, for reporting on drug action and for predicting a favorable therapeutic response. While this science is rapidly emerging at the bench, few epigenomic biomarkers have yet to reach the clinical interface.

Finally, we face a general challenge in the availability of instructive prototype inhibitors. If you ask most disease biologists, they will readily bemoan the lack of potent and selective chemical probes for targets or pathways of interest. Furthermore, when new drug molecules or probes are created, there is typically a lack of immediate and unrestricted availability. I experienced this throughout my own research training. So in our laboratory we have undertaken a social experiment, of sorts, to make chemical probes arising from our research freely and immediately available to research laboratories, importantly without restrictions on use or on amount of compound needed. It is early days in this experiment, but already we have observed an increase in publications around BET bromodomains, many of which utilize the JQ1 chemical probe. We plan to collect more sophisticated data, using relevant literature controls, so please stay tuned. Fundamentally, we believe that a more open-source approach to drug discovery could massively accelerate pre-clinical research timelines and expand the scope of research beyond even our own hypotheses.

Other challenges exist such as a decline in federal funding for research, challenges to efficient collaboration between the biotechnology and pharmaceutical industries, and others, but I firmly believe that this is a time of incredible excitement and optimism. In our field today, there is a palpable sense of progress and impact.

## How do you see this area of drug discovery developing in the next 5 to 10 years?

The next 10 years will surely be marked by an acceleration in epigenomic technology development, the mechanistic dissection of somatic alterations in chromatin-associated factors in cancer, the validation of new context-specific targets, the early understanding of epigenomic drug resistance, a proliferation of epigenomic research beyond cancer (for example, inflammation and cardiovascular disease), the discovery of new small-molecule modulators of chromatin-associated factors and the definitive development of second-wave epigenomic therapies. My greatest hope for our field is that mechanistic insights and clinical science successfully mature these new technologies into breakthrough therapies for patients.

## Abbreviation

IDH2: Isocitrate dehydrogenase 2
